# *Notes from the Field*: Legionellosis Outbreak Associated with a Hotel Aquatics Facility — Tennessee, 2017

**DOI:** 10.15585/mmwr.mm6702a5

**Published:** 2018-01-19

**Authors:** Jane K. Yackley, David Sweat, Mary-Margaret A. Fill, Katie Garman, John R. Dunn

**Affiliations:** ^1^Council of State and Territorial Epidemiologists Applied Epidemiology Fellowship; ^2^Tennessee Department of Health, Division of Communicable and Environmental Diseases and Emergency Preparedness; ^3^Shelby County Public Health Department, Memphis, Tennessee.

On June 26, 2017, the Tennessee Department of Health (TDH) was notified by CDC of two travel-associated cases of legionellosis. The patients resided in Florida and the United Kingdom but had a common hotel exposure in Memphis, Tennessee. On June 27, the Shelby County Health Department identified a third case in a Shelby County resident with the same hotel exposure. All three persons had positive *Legionella* urinary antigen tests and reported using the hotel hot tub. A joint state and local investigation was launched, which included environmental health, epidemiologic, and laboratory components. Shelby County environmental health specialists conducted an assessment of the hotel aquatics facility and identified improper water treatment monitoring and low chlorine residuals (0 ppm; acceptable range = 1–3 ppm). On June 28, TDH was notified of four additional travel companions with illness after exposure to the hotel aquatics facility, including two persons with confirmed *Legionella*, one of whom died.

A public health directive was issued to the hotel on June 28, closing the aquatics facility and requiring consultation with an environmental engineering firm familiar with CDC *Legionella* reduction guidance for the assessment, testing, and remediation of the water distribution systems (*1*). Aquatics area water sampling was also conducted by a TDH environmental health specialist. Laboratory testing of the aquatics facility water samples identified three *Legionella* polymerase chain reaction–positive samples from the pool, pool sand filter, and hot tub sand filter, and isolated *Legionella pneumophila* serogroup 1 from the hot tub sand filter. The remediation firm isolated two nonpneumophila *Legionella* species, including an isolate from the aquatics facility sprinkler system.

An online survey was created to capture epidemiologic and exposure information among hotel guests. A guest roster (including hotel guests before and after the official opening of the aquatics facility on May 27 [i.e., from May 15 to June 27]) was requested. On July 6, approximately 4,000 emails and 209 letters containing the survey link were sent to guests with available contact information. As of July 31, the survey end date, 983 responses were received. Through survey responses, CDC reciprocal notification of non-Tennessee cases, and calls received at the health department, 92 cases were identified, including nine laboratory-confirmed (urinary antigen positive) cases, 19 probable (self-reported pneumonia) cases, and 64 suspected (self-reported fever and ≥1 compatible symptom*) cases. All persons reported hotel stay dates during May 15–June 27. Cases represented persons from 29 states, the United Kingdom, Canada, and Australia. Median age of the persons was 55 years (range = 13–81 years), and 26 (28%) persons reported being a current or former smoker. Sixteen persons were hospitalized, and one aforementioned person died. In a case-control analysis, illness was strongly associated with the aquatics facility (odds ratio = 11.2 [95% confidence interval = 3.4–37.4]).

The incidence of legionellosis has increased approximately 4.5-fold in the United States since 2000, and an increasing number of *Legionella* outbreaks have been reported (*2*,*3*). Hotels are a commonly reported site of *Legionella* outbreaks; however, prompt identification of clusters can be challenging because of the transient nature of the exposed population. Approximately 10% of *Legionella* infections are fatal; therefore, timely investigation of cases is critical (*4*).

This outbreak highlights the importance of rapid case notification and collaboration among environmental health, epidemiologic, and laboratory disciplines during legionellosis outbreaks. In this outbreak, rapid reporting of domestic and international travel-associated cases to CDC and reciprocal notification facilitated rapid identification of the common hotel exposure and initiation of an outbreak investigation, potentially preventing additional morbidity and mortality. Within 3 days of notification, an environmental assessment was performed, and within 12 days, preliminary epidemiologic analyses and laboratory results were available. The combined environmental health, epidemiologic, and laboratory findings helped identify and implicate the hotel aquatics facility.

This outbreak investigation also highlights the need for ongoing health care provider education regarding the importance of obtaining clinical isolates for public health legionellosis investigations. No appropriate clinical specimens were identified or available for culture, which is required for subtyping and comparison with environmental isolates. That 70 (76%) persons reported seeking medical care and 16 (17%) were hospitalized during this outbreak suggests missed opportunities for specimen collection. Provider education around testing methods and the need for clinical isolates during outbreaks could improve future *Legionella* outbreak investigations.
